# Cytotype classification and genetic diversity of *Platostoma palustre* revealed by rDNA localization and chloroplast genome

**DOI:** 10.1186/s12864-025-12118-3

**Published:** 2025-10-21

**Authors:** Chunhui Zhao, Xinyi Li, Xiu Lan, Rupeng Zhao, Ruolan Huang, Lixia Ruan, Zhaoqin Cai, Zhenling Huang, Wanling Wei, Huixian Chen, Hengrui Li, Haixia Yang

**Affiliations:** 1 Guangxi South Subtropical Agricultural Science Research Institute, Guangxi Academy of Agricultural Sciences, Chongzuo, China; 2 Guangxi Academy of Agricultural Sciences, Nanning, 530007 China; 3 State Key Laboratory for Conservation and Utilization of Subtropical Agro-bioresources, College of Life Science and Technology, Guangxi University, Nanning, 530004 China

**Keywords:** Platostoma palustre, Germplasm resources, Fluorescence *in situ* hybridization, Genetic diversity, Chloroplast genome

## Abstract

**Background:**

*Platostoma palustre* A. J. Paton is an edible medicinal plant that plays a significant role in traditional food production and medicinal applications. However, the genetic basis of *P. palustre* remains unclear, thereby hampering research on its genome and polyploid evolution.

**Results:**

To characterize the karyotype and ploidy of *P. palustre*, we performed fluorescence *in situ* hybridization (FISH) by using 35 S and 5 S rDNA probes in *P. palustre*. FISH results indicated that 35 S rDNA mapped to the end of the chromosome (chromosome satellite, heterochromatic region) and that 5 S rDNA was located close to the centromere of the chromosomes. Based on the rDNA sites, we identified three distinct cytotypes of *P. palustre*: diploid (2*n* = 2*x* = 30, *x* = 15), triploid (2*n* = 3*x* = 45, *x* = 15), and tetraploid (2*n* = 4*x* = 60, *x* = 15). To further explore the genetic evolutionary relationship among these *P. palustre* cytotypes, we conducted Illumina sequencing and assembled the chloroplast (CP) genome. The CP genomes of *P. palustre* accessions maintained a conserved single circular molecule with a length of 152,534 − 152,788 bp, comprising a large single-copy region (LSC) and small single-copy region (SSC) separated by two inverted repeat regions (IRs). Phylogenetic trees were also created based on CP and nuclear molecular markers, showing that most *P. palustre* accessions clustered together corresponding to their collection regions. Of these, GDZC2 (2*n* = 2*x* = 30) clustered with several triploid accessions, suggesting that it may share a common ancestor with these triploid accessions.

**Conclusions:**

This is the first study to characterize the karyotype, identify three cytotypes of *P. palustre* using FISH, and provide molecular evidence for an evolutionary relationship among different *P. palustre* accessions. These findings will be useful for further genomic studies and polyploid evolution of *P. palustre*.

**Supplementary Information:**

The online version contains supplementary material available at 10.1186/s12864-025-12118-3.

## Background

*Platostoma palustre* A. J. Paton, also known as *Mesona chinensis* Benth, is a member of the Lamiaceae family and is widely distributed in southern China and Southeast Asian countries. The aerial parts of *P. palustre* are used to produce herbal teas or jelly deserts as medicine or functional food [1, 2]. Thus, *P. palustre* has diverse of applications in herbal beverages, refrigerants, natural edible pigments, coating agents, edible films, and packaging [3–6]. Consequently, the popularity of *P. palustre* has been gradually increasing as it holds substantial potential in the pharmaceutical, healthcare, and food industries. However, the genetic characteristics of *P. palustre*, such as karyotype, ploidy, and evolution, remain largely unknown. Hence, there is an urgent need to study the cytogenetic and genetic diversity to promote the understanding of the polyploid evolution in *P. palustre*.

Knowledge regarding the karyotype and ploidy levels of an organism or cell type presents important piece of information in evolutionary, population and genomic studies. Fluorescence in situ hybridization (FISH) is an efficient cytogenetic tool that allows ploidy and chromosome number determination in plants [7]. Repetitive DNA is a well-known probe that generates specific FISH signal patterns on individual chromosomes for cytogenetic studies in plants [8–10]. In eukaryotes, the most conserved and ubiquitous repetitive DNA is ribosomal DNA, consisting of the major 35 S rDNA that codes for 18 S, 5.8 S and 25 S rRNAs, and minor 5 S rDNA [11, 12]. The numbers of 5 S and 35 S rDNA sites in the genome vary significantly among species, with some species containing up to several thousand copies arranged in tandem arrays, resulting in various chromosomal loci [11, 13]. Therefore, FISH with tandem repeat markers is an essential tool for studying chromosomal structure differentiation, species formation, and evolution.

Chloroplasts (CPs) are important organelles that perform photosynthesis and produce compounds for plant growth. In plants, the CP genome generally ranges in size (120–160 kb) and exhibits a highly conserved structure comprising a single circular DNA molecule with a quadripartite structure that includes an inverted repeat region (IR, such as IRa/IRb) that separates it into one large single-copy region (LSC) and a small single-copy region (SSC) [14, 15]. Compared to the nuclear genome, CP genome is more conserved in terms of structure, gene number and gene composition, and has a relatively moderate evolution rate [16]. The low cost of assembly, small genome size, and high copy number per cell have facilitated CP sequencing in numerous plants [17–21]. Thus, CP genome sequences have been extensively used for phylogenetics analyses and species identification [22, 23]. Additionally, CP genomes show predominantly maternal inheritance, making them suitable for understanding species evolution [24, 25].

The cytogenetic and geographic diversity of *P. palustre* remains unclear, which impedes research on its evolution and genome sequencing. This study aimed to develop an understanding of the karyotype, ploidy, and the relationships among *P. palustre* accessions across different locations. We conducted FISH to examine the distribution of 5 S and 35 S rDNA and the karyotypes of *P. palustre*. We performed next-generation sequencing and assembled the CP genome to explore the phylogenetic relationships in *P. palustre* across various geographic localities. This study will provide useful information for understanding the cytogenetic base and genetic diversity of this species and will lay a foundation for further genome sequencing and exploring the evolution of different ploidies in *P. palustre*.

## Methods

### Plant materials

Thirty *P. palustre* accessions were used in the present study (Table 1). These samples were collected from seven locations: two accessions from Jiangxi, seven from Fujian, seven from Guangxi, 11 from Guangxi, one from Anhui, one from Taiwan and one from Vietnam. The cutting seedlings of *P. palustre* were planted in a germplasm resource nursery at the Guangxi South Subtropical Agricultural Science Research Institute. Young leaves were used for the genomic DNA extraction to perform whole genome sequencing (WGS), using the cetyltrimethyl ammonium bromide (CTAB) procedure [26]. A distribution map was constructed using Chitplot (https://www.chiplot.online/).

**Table 1:** The experimental materials used in this study

Accessions	Locations	Latitude and longitude	Accessions	Locations	Latitude and longitude
Jiangxijian (JXJA)	Jiangxi, China	23.57 N,116.36E	Guangxipingnan4 (GXPN4)	Guangxi, China	24.26 N,116.13E
Jiangxijian2 (JXJA2)	Jiangxi, China	23.57 N,116.36E	Guangdongmengzhou (GDMZ)	Guangdong, China	25.00 N,121.58E
Fujianshishenmiao5 (FJSSM5)	Fujian, China	25.08 N,117.03E	Guangdongzengcheng1 (GDZC1)	Guangdong, China	23.81 N,110.25E
Fujiancao (FJC)	Fujian, China	25.97 N,119.4E	Guangdongzengcheng2 (GDZC2)	Guangdong, China	22.32 N,109.11E
Fujianxiabei4 (FJXB4)	Fujian, China	22.42 N,111.04E	Guangdongmaoming1 (GDMM1)	Guangdong, China	22.42 N,111.043E
Fujianlingyi (FJLY)	Fujian, China	25.08 N,117.03E	Guangdongmaoming3 (GDMM3)	Guangdong, China	25.06 N,116.08E
Fujianxiabei (FJXB)	Fujian, China	24.89 N,116.07E	Guangdongmaoming7 (GDMM7)	Guangdong, China	23.15 N,113.63E
Fujianshishenmiao3 (FJSS3)	Fujian, China	26.72 N,106.93E	Guangdongmaoming2 (GDMM2)	Guangdong, China	22.42 N,111.04E
Fujianwuping (FJWP)	Fujian, China	22.42 N,111.04E	Guangdongmaoming4 (GDMM4)	Guangdong, China	22.42 N,111.04E
Guangxijiangxia (GXJX)	Guangxi, China	23.91 N,110.25E	Guangdongmaoming6 (GDMM6)	Guangdong, China	25.08 N,117.03E
Guangxiwuzhou(GXWZ)	Guangxi, China	23.44 N,111.27E	Guangdongmaoming5 (GDMM5)	Guangdong, China	22.42 N,111.04E
Guangxipubeijiangping (GXPBJP)	Guangxi, China	24.78 N,113.59E	Guangdongcao (GDC)	Guangdong, China	23.11 N,113.33E
Guangxipingnan1 (GXPN1)	Guangxi, China	23.81 N,110.25E	Anhuibaimao (AHBM)	Anhui, China	30.82 N,115.95E
Guangxilingshan (GXLS)	Guangxi, China	23.15 N,113.63E	Taiwancao (TWC)	Taiwan, China	23.57 N,116.36E
Guangxipingnan2 (GXPN2)	Guangxi, China	23.81 N,110.25E	Yuenancao (YNC)	Vietnam	21.01 N,105.53E

### Chromosome preparation and fluorescent in situ hybridization (FISH)

Root tips were treated with para-dichlorobenzene and α-bromonaphthalene at room temperature (RT, 25 °C) for 4.5 h, then collected and fixed in Carnoy’s (alcohol: acetic acid = 3:1) fixative solution for 12 h. Using the cell wall-degrading enzyme (1% pectolyase Y23, 2% pectinase, 2% RS and 4% cellulase Onozuka R-10), the root tips were digested for 2 h at 37 ℃. The cell suspension was prepared and dropped onto a glass slide and fixed with 10 µL Carnoy’s fixative solution.

The 35S rDNA probe was derived from the pTA71 plasmids from wheat [27], and was labelled using digoxigenin (Dig)−11-dUTP through nick translation (Roche, USA). 5S rDNA was amplified using two primers (5S-U: 5’‐TCCTGGGAAGTCCTCGTGTTGCAT‐3’ and 5 S‐L: 5’‐GGTCACCCATCCTAGTACTACTCT‐3’) [28]. Polymerase chain reaction (PCR) products were purified and labelled by nick translation using biotin-dUTP. FISH was performed based on the published protocols [29], with minor adjustments. Before FISH, slides containing metaphase cells were incubated at room temperature for 24 h, followed by denaturation on a heating block at 70 °C for 3 min. Slides were incubated at 37℃ overnight in a humid dark box, and then washed for 5 min in 2×SSC (RT), for 10 min in 2×SSC (RT), and 3 min in 1×PBS (RT). Digoxigenin and biotin-labeled probes were detected using rhodamine anti-Dig-sheep (Roche, USA) and Alexa Fluor 488 Streptavidin (Life Technologies, USA), respectively. FISH images of separate channels were captured using an CDD camera of Olympus BX51. Images were processed and cropped using Photoshop and Image J. DRAWID software [30] was used to measure individual chromosome length for karyotyping. Cytological and karyotyping analyses were performed in 10 metaphase cells from each *P. palustre* accession.

### CP genome assembly and annotation

Thirty *P. palustre* accessions were sent for next generation sequencing (NGS) by Benagen Tech Solutions. An Illumina paired-end library was used for NovaSeq 6000 sequencing, achieving approximately 2.35–3.93× genome coverage with the *Mentha longifolia* CMEN585 genome as a reference (GCA_001642375.2) (Table S2). The CP genome was de novo assembled using GetOrganelle software (https://github.com/Kinggerm/GetOrganelle). CP genes were visualized by using the online CpGAVAS platform [31], and default parameters were used to predict PCGs, tRNAs, and rRNAs. OrganellarGenomeDRAW software [32] was used to map the CP genome.

### Comparative CP genome analysis and phylogenetic tree construction

Because 16 *P. palustre* accessions had identical CP genome sequences compared to other accessions, we selected the 14 unique cpDNA genomes for further analysis. The mVISTA program was used in the Shuffle-LAGAN mode [33] to detect variations within CP genomes. Next, the boundaries between the IR and SC (contraction/expansion) regions were also compared and analyzed using the IRScope online site (https://irscope.shinyapps.io/irapp/). The diploid FJC was used as a reference using the mVISTA software. A sliding window method was used to analyze nucleotide variability using DnaSP software (version 5.1.0). The entire CP genome of 14 *P. palustre* accessions was aligned using MAFFT7.0 (https://mafft.cbrc.jp/alignment/software/), with *Stachys byzantina* (NC029825) and *Pogostemon cablin* (MF287373) as outgroups. The phylogenetic analysis was performed based on maximum likelihood (ML) by IQ-TREE multicore 2.3.4 (http://www.iqtree.org/). IQ-TREE was also used to find the optimal model, and the transitional substitution model with three substitution types, empirical base frequencies, and freeRate heterogeneity (TIM3 + F + R4) was selected for the phylogenetic analysis. The bootstrap probability of each branch was calculated with 1000 replications, and the rooted phylogenetic tree was visualized using the web application tvBOT (version 2.6.1).

### Variants calling, phylogenetic tree construction, and principal component analysis (PCA)

The clean paired-end reads of *P. palustre* accessions were aligned to *Mentha longifolia* CMEN585 genome (GCA_001642375.2) using bwa-mem2 (version 2.2.1) with default parameters, followed by duplicate marking with samblaster (version 0.1.26) and conversion of SAM files to BAM files using samtools (v1.21). Then, each sample was analyzed by GATK (version 4.6.0.0) HaplotypeCaller with --ERC GVCF, after which single nucleotide polymorphisms (SNPs) were extracted using bcftools view (version 1.21; -v snps) and filtered with GATK VariantFiltration using stringent quality thresholds (QUAL < 30.0, QD < 2.0, MQ < 40.0, FS > 60.0, SOR > 3.0, MQRankSum <−12.5, ReadPosRankSum <−8.0). The distance matrix was calculated using the VCF2Dis software (https://github.com/BGI-shenzhen/VCF2Dis), and the phylogenetic tree was visualized using the web application tvBOT (version 2.6.1). The filtered SNPs were simultaneously used to PCA analysis in Plink (version 1.90b6.21) with default parameters, and the results were visualized through three-dimensional (3D) plots using the R package scatterplot3d to elucidate the genetic structure.

## Results

### Chromosome counting

*P. palustre* is an important edible medicinal plant. However, the basic genetic information of *P. palustre* remains unknown, which affects genomic research and germplasm innovation. We collected 30 *P. palustre* accessions that come from seven different locations: Jiangxi, Fujian, Guangxi, Guangxi, Anhui, Taiwan and Vietnam (Table 1). Therefore, we first aimed to identify the exact cytotype of *P. palustre* using FISH. We obtained chromosome preparations suitable for counting the chromosomes in 30 *P. palustre* accessions, and observed three different chromosome numbers. This suggests that *P. palustre* has three cytotypes: 2*n* = 30, 2*n* = 45 and 2*n* = 60 (Table S1).

### Chromosomal rDNA location and karyotyping

**Fig. 1:**
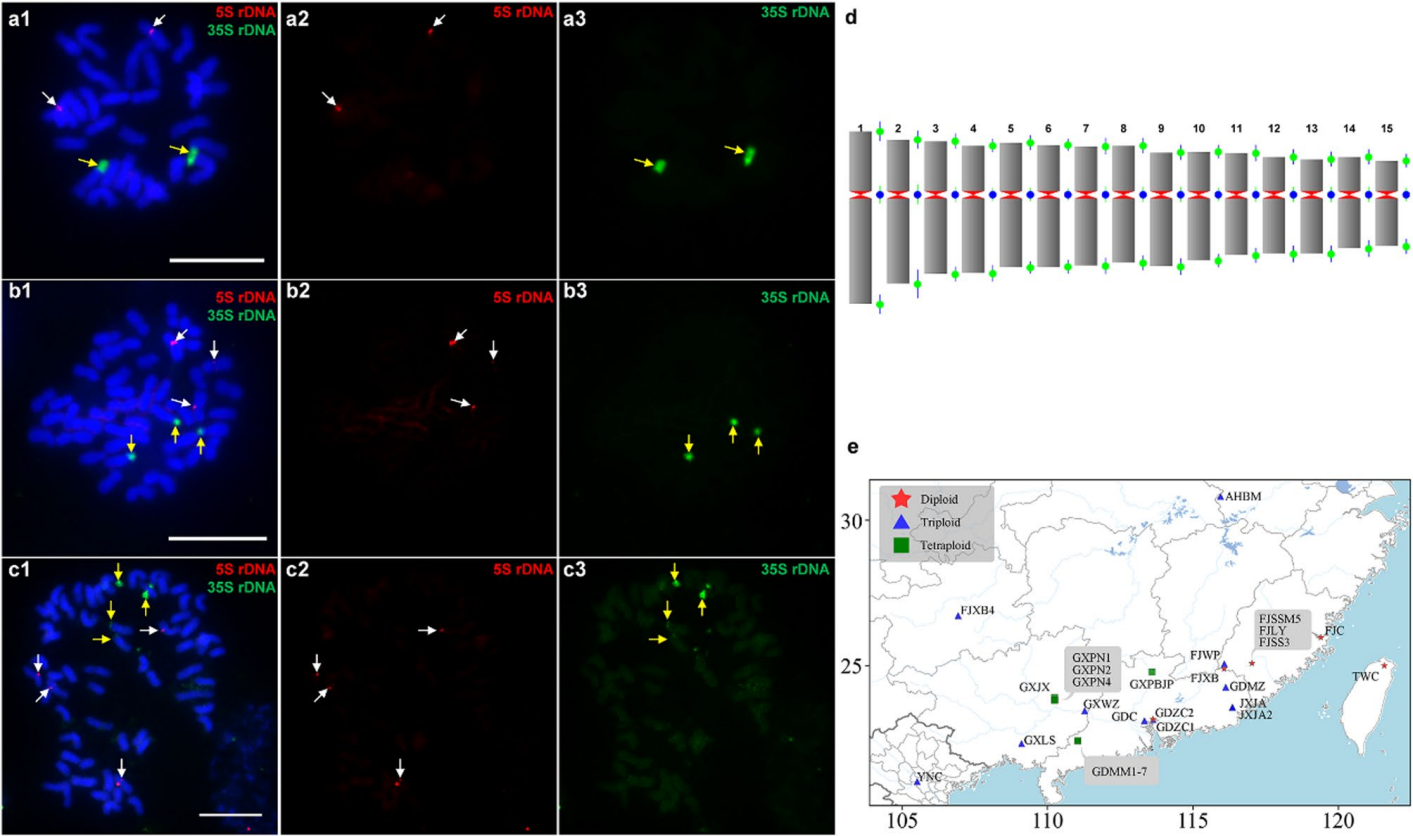
Cytogenetic analysis of 5 S and 35 S rDNAs on mitotic metaphase chromosomes of *P. palustre*. **a-c** The 5 S rDNA signals appear red and are indicated using white arrows. 5 S rDNA was close to the centromere of the chromosome and appeared in two, three, and four loci in diploid FIC (**a**), triploid FJXB4 (**b**), and tetraploid GXNP4 (**c**) respectively. 35 S rDNA signals appear green and are indicated using yellow arrows. 35 S rDNA was in the subterminal region of the chromosome and appeared in two, three and four loci in FIC (**a**), FJXB4 (**b**), and GXNP4 (**c**) respectively. Scale bars = 10 μm. **d** Karyotype of the diploid *P. palustre* FJC. Vertical green dots indicated standard deviations (SD) of chromosome arm lengths and vertical blue dots indicated SD of chromosome centromere indices. **e** Distribution of different *P. palustre* accessions

Whether chromosomal location of the rDNAs is conserved in these different *P. palustre* accessions remains unknown. Thus, we conducted FISH assays using 5 S and 35 S rDNA as probes to produce red and green signals, respectively (Fig. 1a-c). For 5 S rDNA, we detected two, three and four loci in the experimental *P. palustre* accessions (Table S1, Fig. S1). All 5 S rDNA locations were close to the centromere of the chromosomes of *P. palustre* (Fig. 1). A similar pattern was observed for 35 S rDNA, with locus numbers corresponding to 5 S rDNA and ploidy level (Table S1, Fig. S1). However, the 35 S rDNA was located at subterminal regions of chromosomes (Fig. 1a). Altogether, these results indicate that 5 S and 35 S rDNAs are highly conserved in different *P. palustre* accessions, and *P. palustre* contained diploid, triploid, and tetraploid, with a basic chromosome number *x* = 15.

In summary, diploid and triploid *P. palustre* accessions were distributed in Fujian, triploid accessions in Jiangxi, and triploid and tetraploid accessions occurred in Guangxi (Fig. 1e). However, all diploid, triploid and tetraploid accessions were occurred in Guangdong, implying that frequent polyploidization events may have occurred in this region (Fig. 1e). Additionally, we also constructed the karyotype for the diploid *P. palustre* FJC (Fig. 1d), all chromosomes were measured and ranged 2.62–5.52 micrometers (µm) in size (Table 2). Twelve chromosomes are metacentric with the arm ratio ranged 1.40–1.67, whereas the remaining chromosomes are submetacentric with an arm ratio ranged 1.77–1.80 (Fig. 1d; Table 2).

**Table 2:** Arm ratio and lengths of individual chromosomes in *P. palustre* FJC

Chromosome	Chromosome length (µm)	Long arm Length (µm)	Short arm Length (µm)	Arm rato^a^
1	5.52 *±* 0.12	3.53 *±* 0.18	1.98 *±* 0.18	1.80 *±* 0.25
2	4.57 *±* 0.25	2.85 *±* 0.33	1.72 *±* 0.16	1.68 *±* 0.31
3	4.21 *±* 0.06	2.53 *±* 0.11	1.68 *±* 0.13	1.53 *±* 0.18
4	4.02 *±* 0.09	2.50 *±* 0.13	1.52 *±* 0.08	1.66 *±* 0.16
5	3.93 *±* 0.07	2.31 *±* 0.07	1.62 *±* 0.10	1.43 *±* 0.13
6	3.83 *±* 0.06	2.29 *±* 0.09	1.55 *±* 0.06	1.48 *±* 0.11
7	3.76 *±* 0.09	2.26 *±* 0.13	1.50 *±* 0.14	1.52 *±* 0.21
8	3.67 *±* 0.10	2.15 *±* 0.12	1.52 *±* 0.12	1.42 *±* 0.16
9	3.56 *±* 0.07	2.27 *±* 0.14	1.29 *±* 0.10	1.77 *±* 0.26
10	3.39 *±* 0.05	2.07 *±* 0.08	1.32 *±* 0.10	1.58 *±* 0.17
11	3.16 *±* 0.12	1.89 *±* 0.12	1.27 *±* 0.15	1.52 *±* 0.25
12	3.00 *±* 0.08	1.86 *±* 0.15	1.14 *±* 0.11	1.66 *±* 0.30
13	2.92 *±* 0.06	1.85 *±* 0.15	1.07 *±* 0.11	1.77 *±* 0.34
14	2.82 *±* 0.12	1.68 *±* 0.14	1.14 *±* 0.12	1.49 *±* 0.25
15	2.62 *±* 0.07	1.59 *±* 0.12	1.03 *±* 0.08	1.57 *±* 0.24

^a^Arm ratio, length of the long arm/length of the short arm

### CP genome assembly and annotation

**Fig. 2:**
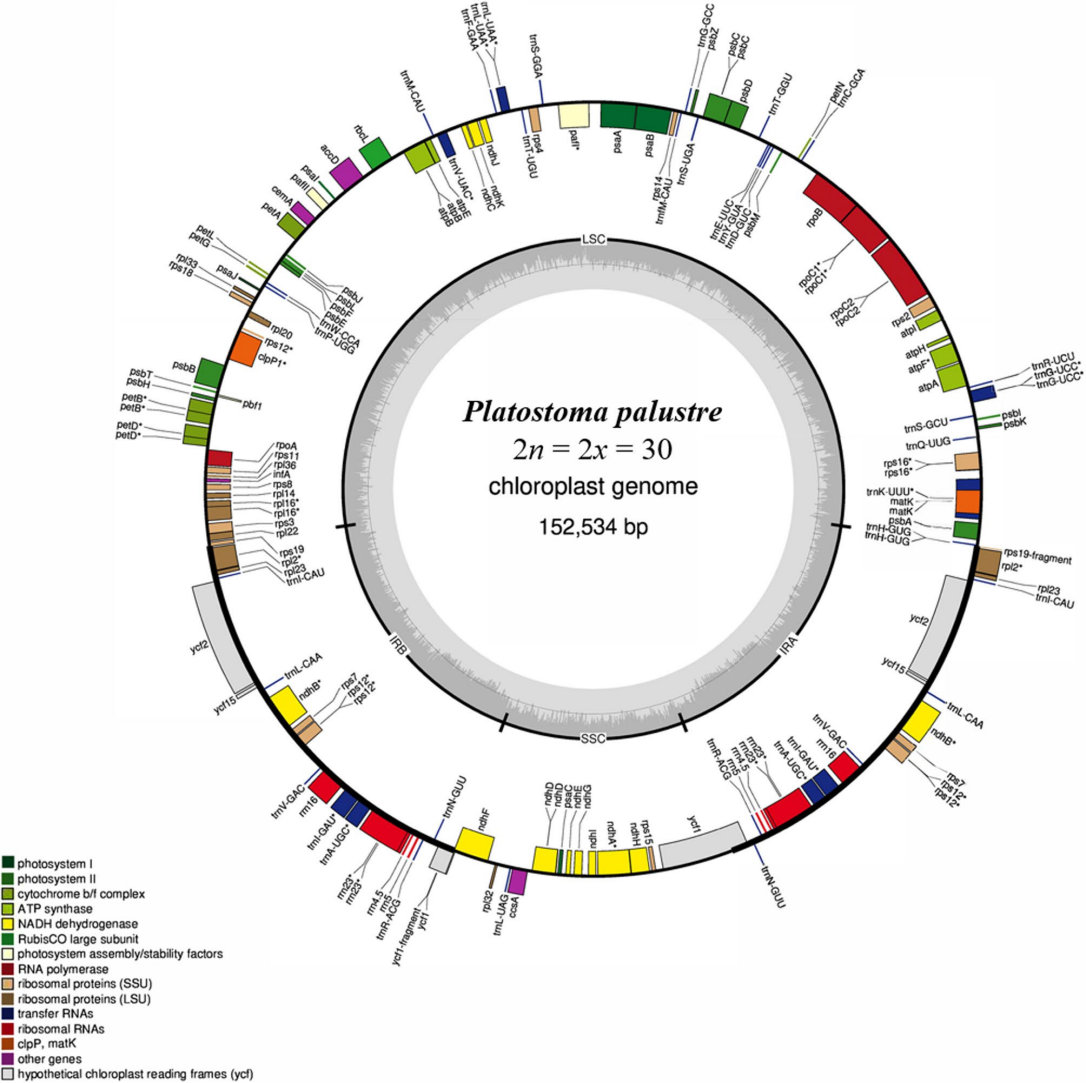
A schematic depiction of the CP genome of *Platostoma palustre* FJC. The map contains four parts: a large single-copy region (LSC), small single-copy region (SSC) and two inverted repeat regions (IRs). Genes are annotated with different colors based on their functional classifications

The genomic DNA of *P. palustre* was sequenced using the Illumina platform (HiSeq 4000), and approximately 12.40 ~ 19.26 G clean data were generated (Table S2). The GC content, Q20, and Q30 values of clean data were 39.75–41.12%, 98.27–98.51%, and 93.34–94.77%, respectively (Table S2). The nucleotide sequences of 30 *P. palustre* CP genomes were assembled into a single circular molecule with a total length of 152,534 − 152,788 bp (Fig. 2 and Table S3). We analyzed the basic characteristics of the *P. palustre* CP genome. All 30 CP genomes shared a typical quadripartite CP structure that comprised a pair of inverted repeats (IR) regions of 51,336 − 51,774 bp; a large single-copy (LSC) region of 83,450 − 83,522 bp; and a small single copy (SSC) region of 17,725 ~ 17,776 bp (Fig. 2, Table S3). Based on the CP genome sequence consistency, *P. palustre* was divided into 14 groups (Table S3). Of these, the diploid accessions (groups I and XIII) presented the shortest CP length (152,534 bp), whereas the triploid accessions (group XI) were the longest (152,778 bp) (Table S3). Annotation of the CP genome of *P. palustre* indicated that it encodes 129 unique genes, including 84 protein-coding genes (PCGs), 37 transfer RNAs (tRNAs), and eight ribosomal RNAs (rRNAs) (Table S4).

### Sequence variation and CP genome junction characteristics

**Fig. 3:**
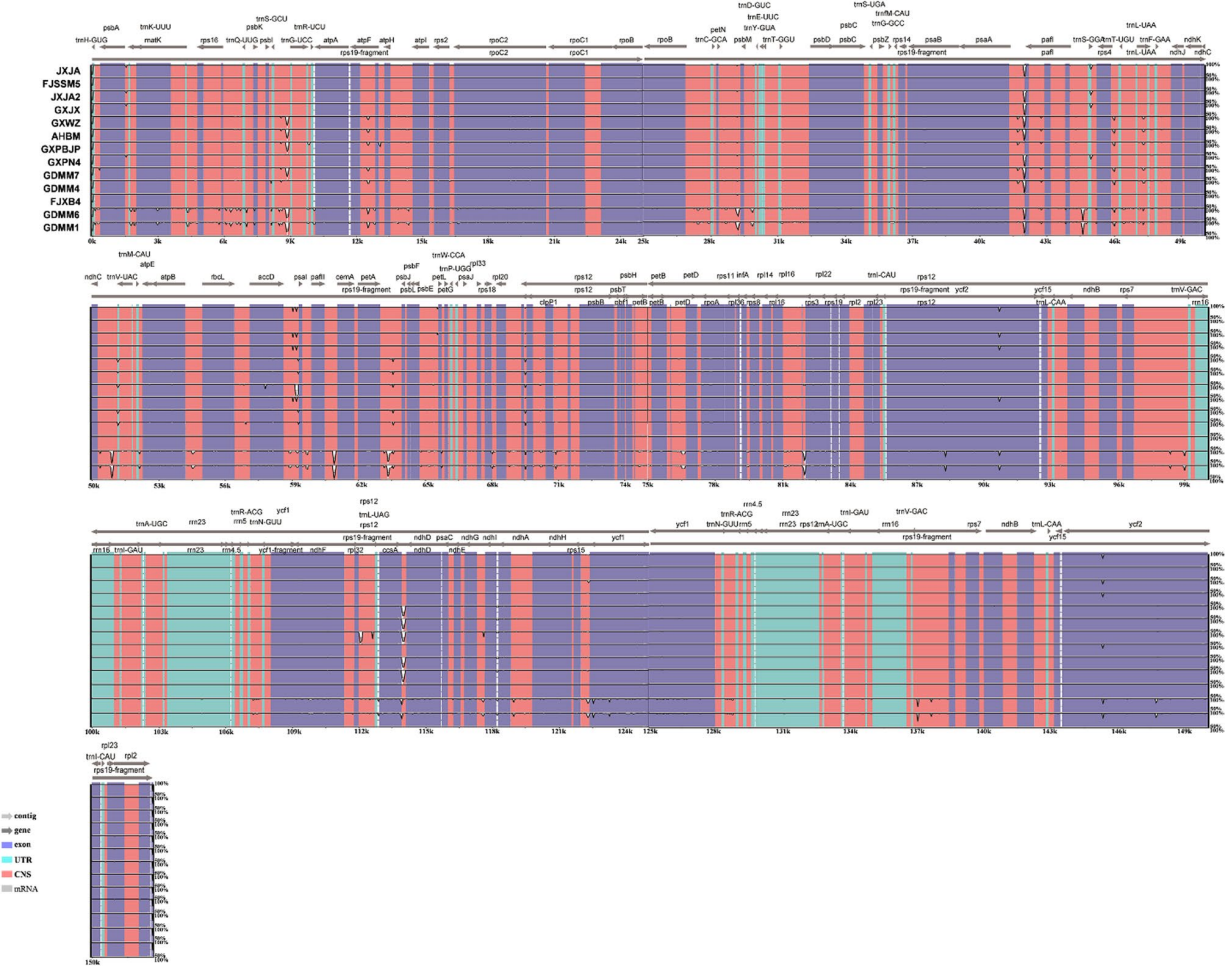
Sequence identity plot comparing the *Platostoma palustre* chloroplast (CP) genomes with FJC as a reference. Gray arrows and thick black lines above the alignment indicate genes with their orientation. Purple bars represent exons, sky-blue bars represent transfer RNA (tRNA) and ribosomal RNA (rRNA), and red bars represent non-coding sequences (CNS). The y-axis represents the identity percentage ranging 50–100%

**Fig. 4:**
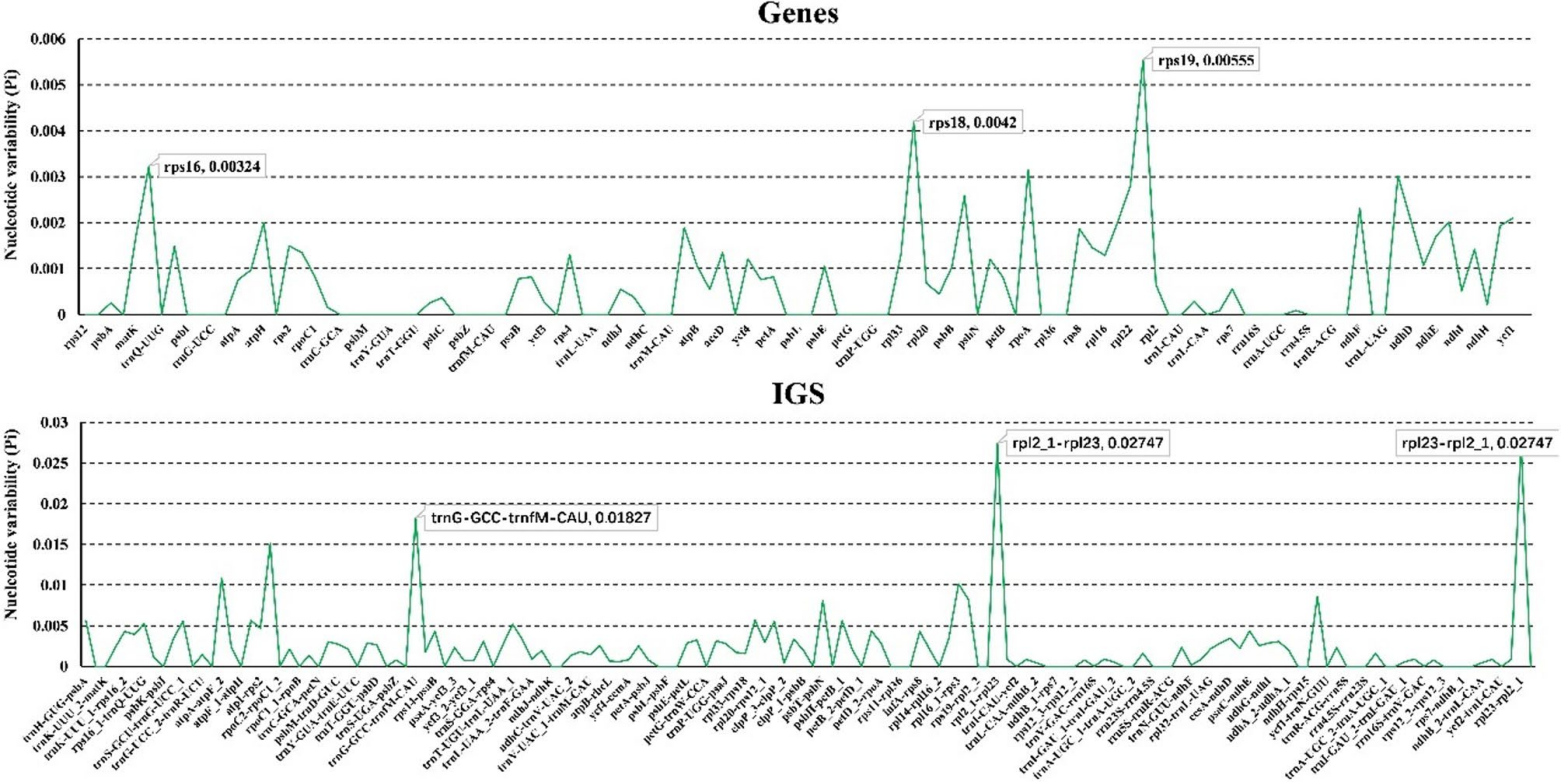
Nucleotide variability analyses of the CP genomes of *Platostoma palustre*. The X-axis notes the name of genes or intergenic spacer (IGS). The Y-axis notes the nucleotide variability value

Considering the identical sequences, 14 complete CP genomes of *P. palustre* accessions were selected for further analysis. The coding regions showed less divergence than the non-coding regions and that the IR regions were more conserved than the LSC and SSC regions (Fig. 3). In the gene regions, the most variable gene was *rps19* (Pi = 0.00555), followed by *rps18* (Pi = 0.0042) and *rps16* (Pi = 0.00324) (Fig. 4). Highly divergent regions were primarily located in the intergenic regions, such as *rpl2_1-rpl23* (Pi = 0.02747), *rpl23-rpl2_1* (Pi = 0.02747) and *trnG-trnfM* (Pi = 0.01827) (Fig. 4). We also analyzed the SNP sites of cp. in the above *P. palustre* accessions with FJC as a reference (Fig. S2). Results showed significant SNP variations at positions ~ 19,177 bp and ~ 22,632 bp (Fig. S2a), and the most pronounced base substitution was the transformation of G to A (Fig. S2b).

**Fig. 5:**
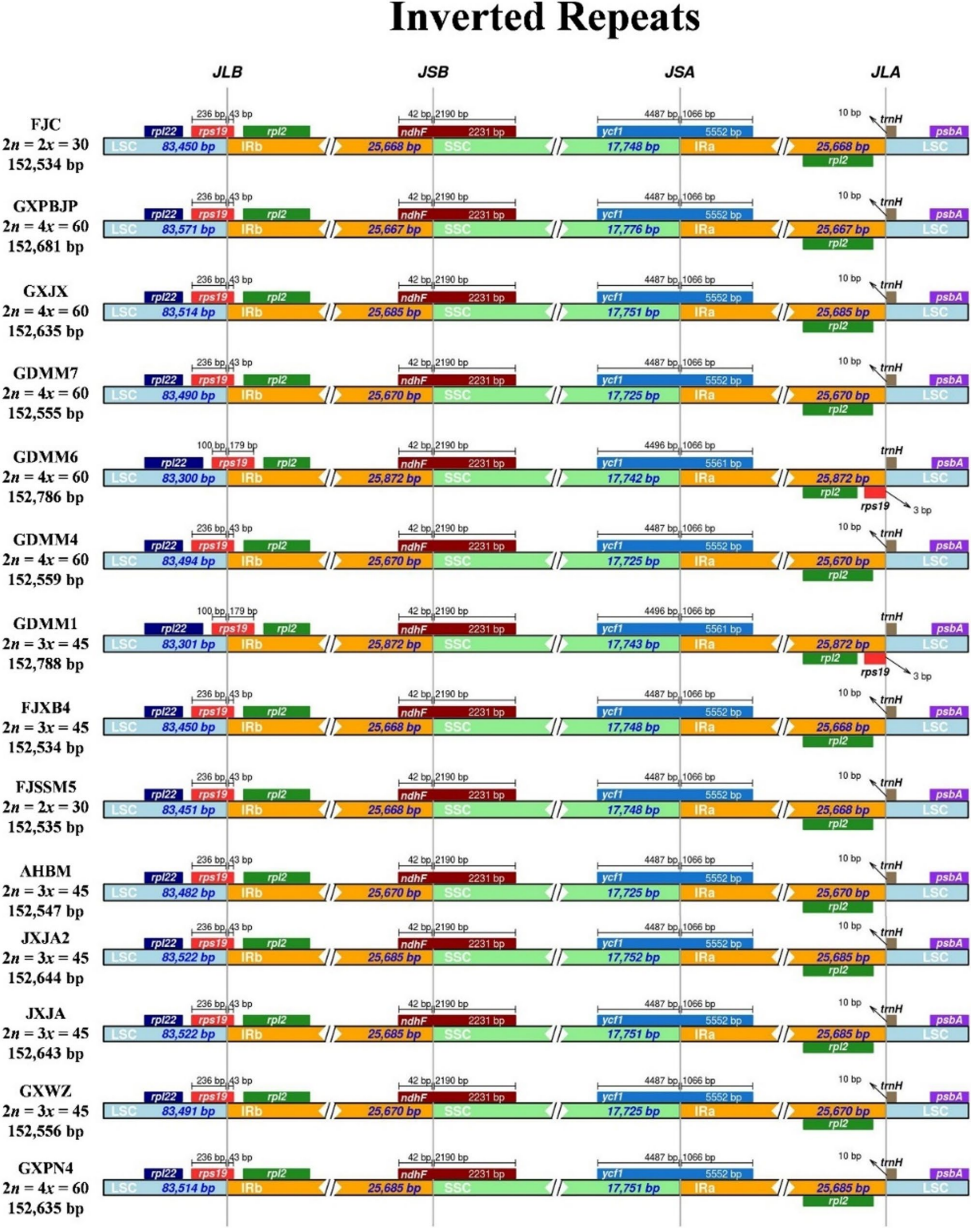
Comparison of the borders of LSC, SSC, and IR regions among the *Platostoma palustre* CP genomes using FJC as the reference

The expansion and contraction of the border regions between the single-copy regions and the two IR regions lead to genome size differences among plant lineages [34]. In this study, the exact IR border positions and their adjacent genes were compared with the diploid FJC CP genome. The *rps19*, *ndhF*, *ycf1*, and *trnH* genes were located at the junctions of the LSC/IRb, IRb/SSC, SSC/IRa, and IRa/LSC regions (Fig. 5). The *rps19* gene was detected at the junction of the LSC and IRb in most accessions, except for GDMM6 (2*n* = 4*x* = 60) and GDMM1 (2*n* = 3*x* = 45), which were also located in the IRa region (Fig. 5). This distinct sequence expansion of *rps19* in the IR region resulted in a larger CP genome than that of the other *P. palustre* accessions (Fig. 5, Table S3). *ndhF* is located at the IRb/LSC junction in all CP genomes. The *ycf1* gene spans the JSA (SSC/IRa) region, which also reflects changes in the JSA region. GDMM6 (2*n* = 4*x* = 60) and GDMM1 (2*n* = 3*x* = 45) had a long *ycf1* sequence of 5,561 bp, whereas the others possessed a sequence of 5,552 bp (Fig. 5). *TrnH* was observed in the LSC region in all 14 CP genomes, three to ten bp away from the junction of IRa/LSC (Fig. 5).

### Phylogenetic and principal component analysis (PCA)

**Fig. 6:**
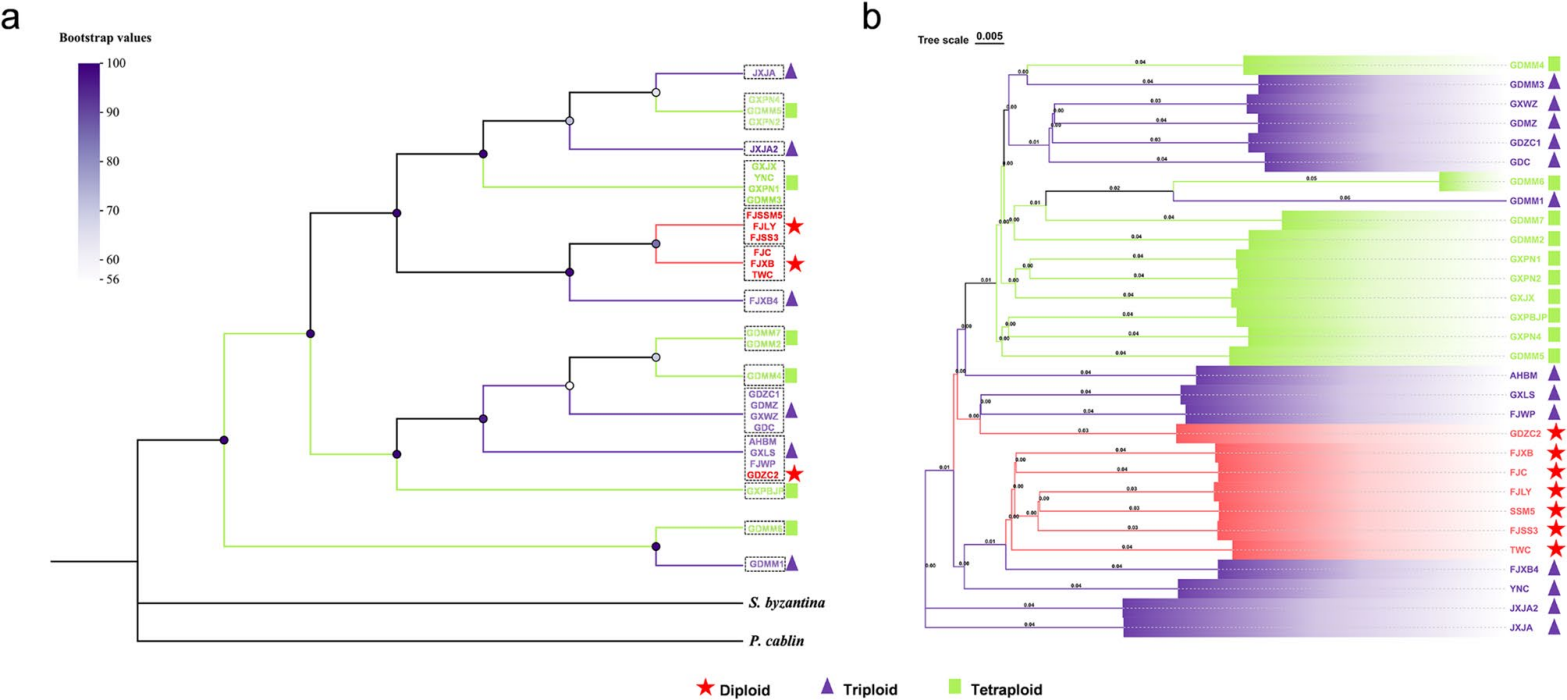
Molecular phylogenetic analysis of P. palustre. **a** Phylogenetic tree based on chloroplast (CP) genome sequences from different *P. palustre* accessions, with *S. byzantina* and *P. cablin* as outgroups. The dotted-line box highlights accessions sharing identical CP genome sequences. Node color indicated bootstrap values. Branch colors correspond to the ploidy of each *P. palustre* accession. **b** Phylogenetic tree based on genome-wide single nucleotide polymorphisms (SNPs) in 30 *P. palustre* accessions. Branch colors correspond to the ploidy of each accession

CP genome phylogenetic analysis plays a crucial role in tracking numerous lineages within plants [35], and the maternal inheritance of CP will help us to understand its origin and evolution. To confirm the evolutionary positions of the different *P. palustre* accessions, 14 CP genomes were selected for evolutionary construction using the maximum likelihood (ML) method. The Results showed that these accessions were divided into several clades consistent with the collection regions (Fig. 6a). Most diploid accessions clustered into two branches distributed in Fujian and Taiwan (Fig. 6a). Diploid GDZC2 was closed to several triploid accessions (AHBM, GXLS, and FJWP), suggesting that these accessions may share a common ancestor (Fig. 6a). These results were also confirmed by another phylogenetic tree constructed based on the SNP of the nuclear genome (Fig. 6b). However, the phylogenies based on cp. and nuclear SNP were not entirely congruent. This discordance is likely caused by the cp. genome representing a predominantly maternally inherited organelle, whereas nuclear loci collectively reflect the genomic signal of biparental inheritance.

**Fig. 7:**
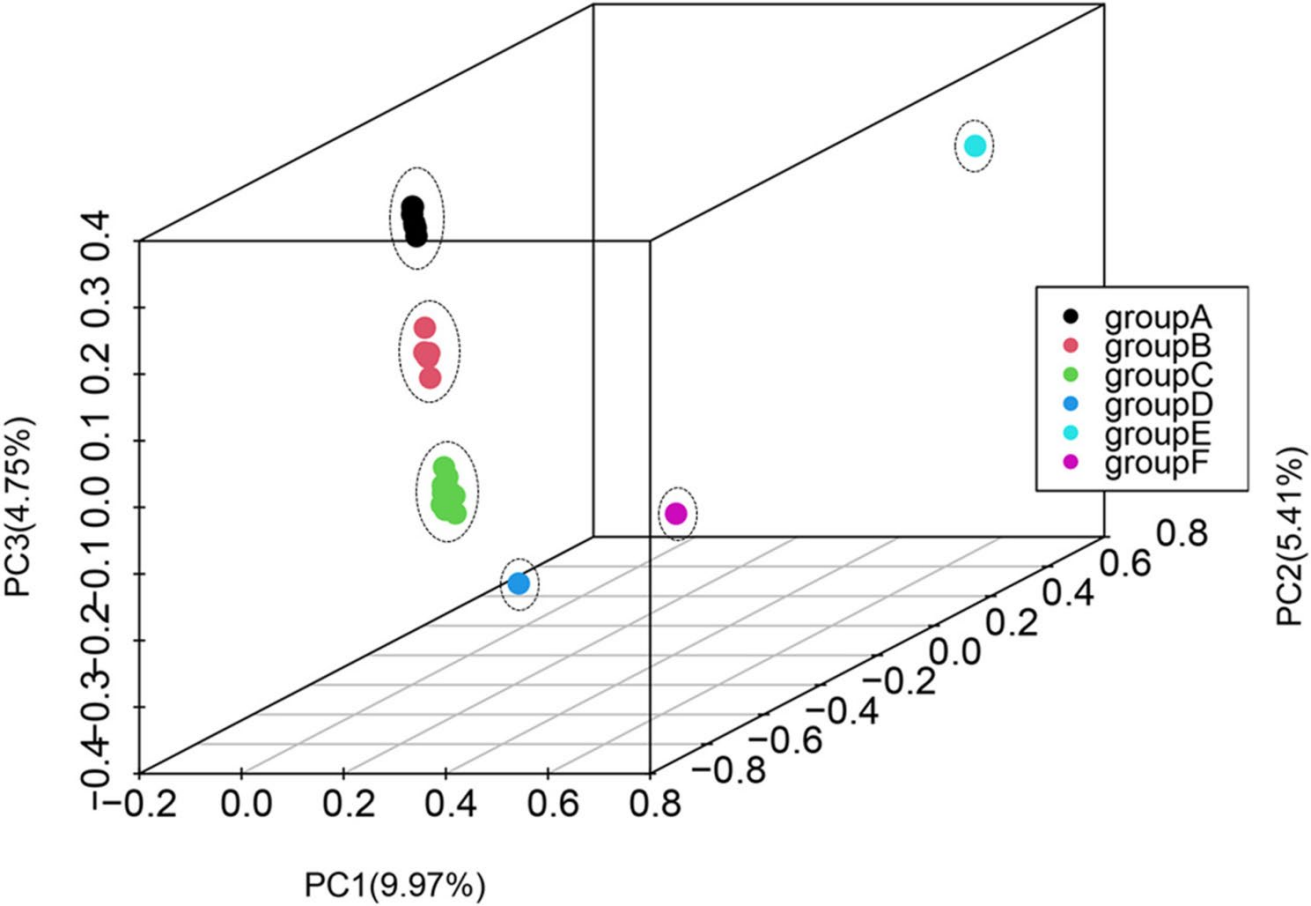
Principal components analysis (PCA) based on nuclear SNPs in *P. palustre*. The variance of the three axes was 9.97%, 5.41% and 4.75%, respectively

The PCA was conducted for nuclear SNPs variation on the three PC axes, and 30 *P. palustre* accessions were divided into six groups, which explained the variance of the three axes at 9.97%, 5.41% and 24.75% (Fig. 7; Table 3). Most diploid accessions were divided to group A, along with one triploid accession (FJXB4) (Table 3). This result indicated that the triploid (FJXB4) has a relatively closed relationship with these diploid accessions compared to the other triploid accessions (Fig. 6a and b). In addition, we determined that one diploid accession GDZC2, was clustered into group B, which included six triploid accessions, revealing that these triploid accessions have a closed relationship with GDZC2 **(**Table 3**)**, which was also consistent with the phylogenetic tree based on the CP and nuclear genome (Fig. 6a and b).

**Table 3:** Principal components analysis cluster statistics of different *P. palustre* accessions

Accessions	Group	Chromosomes	Accessions	Chromosomes	Group	Accessions	Chromosomes	Group
FJSSM5	A	2*n* = 2*x* = 30	YNC	2*n* = 3*x* = 45	B	GDMZ	2*n* = 3*x* = 45	C
FJC	A	2*n* = 2*x* = 30	GXLS	2*n* = 3*x* = 45	B	GXPN4	2*n* = 4*x* = 60	C
FJLY	A	2*n* = 2*x* = 30	GDZC2	2*n* = 2*x* = 30	B	GDZC1	2*n* = 3*x* = 45	C
FJXB	A	2*n* = 2*x* = 30	FJWP	2*n* = 3*x* = 45	B	GDMM4	2*n* = 4*x* = 60	C
FJXB4	A	2*n* = 3*x* = 45	GXJX	2*n* = 4*x* = 60	C	GDMM5	2*n* = 4*x* = 60	C
FJSS3	A	2*n* = 2*x* = 30	GXWZ	2*n* = 3*x* = 45	C	GDMM3	2*n* = 3*x* = 45	C
TWC	A	2*n* = 2*x* = 30	GXPBJP	2*n* = 4*x* = 60	C	GDMM2	2*n* = 4*x* = 60	C
JXJA	B	2*n* = 3*x* = 45	GXPN1	2*n* = 4*x* = 60	C	GDMM7	2*n* = 4*x* = 60	D
JXJA2	B	2*n* = 3*x* = 45	GXPN2	2*n* = 4*x* = 60	C	GDMM6	2*n* = 4*x* = 60	E
AHBM	B	2*n* = 3*x* = 45	GDC	2*n* = 3*x* = 45	C	GDMM1	2*n* = 3*x* = 45	F

## Discussion

The number and appearance of all chromosomes in eukaryotic species is termed as the karyotype. The karyotype provides basic genomic information, and may contribute to understanding phylogenetic relationships and evolutionary origins of the related species. For example, karyotype analysis had revealed the chromosome evolution in *Populus* and *Saccharum spontaneum* [36, 37]. Thus, establishing the karyotypes is important for future plant genomic research. *P. palustre* is an economical plant that is widely used in medicine and food. However, its basic cytogenetic information is largely unknown. To the best of our knowledge, this is the first study to construct a karyotype of *P. palustre* and determine that *P. palustre* contained three cytotypes, 2*n* = 30, 45, and 60 (Figs. 1a-c). Karyotype analysis indicated that the chromosomes of *P. palustre* were metacentric (1.01 < arm ratio < 1.70) [38] or submetacentric (Fig. 1d; Table 2), suggesting that this species has evolved highly.

In cytogenetic studies, repetitive sequences are frequently used as markers to detect the ploidy and perform karyotype analyses in plants, especially 5 S and 35 S rDNAs [39–42]. Generally, the number of rDNA loci in most plants should be consistent with their ploidy. Hence, 35 S and 5 S rDNA have been widely used as cytogenetic markers for ploidy identification and evolution in plants. However, variability in the chromosomal locations of rDNAs has been reported in several species [34–36]. Previous studies have proved that the number of 35 S rDNA loci may deviate from chromosomal ploidy due to the dynamic evolution of 35 S rDNA sequences that could not be used for determining the ploidy [39, 43]. Because the possible instability of the 35 S rDNA loci, 5 S rDNA may be conserved and appropriated for defining ploidy in a species. In our study, we detected the same loci between 5 S and 35 S rDNA in all cytotypes of *P. palustre* (Fig. 1). This suggests that 5 S and 35 S rDNA are powerful markers for detecting the ploidy in *P. palustre*. In most plants, 35 S rDNA is located at the subterminal regions of chromosomes, whereas 5 S rDNA is typically situated near the centromeres [44, 45]. In the present study, we also showed that 5 S and 35 S rDNA mapped to the pericentromeric and subterminal regions, respectively (Fig. 1, Table S1). Therefore, 5 S and 35 S rDNA are evolutionarily conserved in *P. palustre*. Altogether, chromosome counts indicate three distinct ploidy levels (*x* = 15), and rDNA locus number correlates with the ploidy. Although this correlation is observed in *P. palustre*, it is not always conserved across taxa. Thus, this consistent rDNA loci and ploidy levels may suggest autopolyploidy and a recent polyploid origin of *P. palustre*.

The CP is primarily inherited from the maternal parents. Typically, CP adopt a circular molecular structure with a length ranging 120–220 kb and comprised of a LSC region, SSC region, and two IR regions [46]. In this study, the CP genome of *P. palustre* was successfully assembled into a circular molecular structure, and its size, gene count, and quadripartite structure aligned with the fundamental characteristics of plant CP genomes (Fig. 2, Table S3). In addition, we determined that the intergenic regions have higher divergence rates than others based on a comparative analysis among *P. palustre* and its relatives, which is consistent with the findings of a previous study [47]. IR regions are highly conserved and play a pivotal role in stabilizing the CP genome structure [48]. In the present study, the structure of the *P. palustre* CP genome, featuring a pair of IR regions separated by SSC and LSC regions (Fig. 5), is similar to that of most sequenced angiosperm CP genomes. The contraction and expansion of IR regions, a common occurrence in chloroplast genome evolution, may contribute to variations in genome length [49]. Here, we also detected the expansion of *rps19* in IRa region of *P. palustre* GDMM6 and GDMM1, which led to the longest CP length (Fig. 5). CP genome sequencing has provided rich nucleotide sequence data, displaying significant variations both within and between species. This information has been instrumental in addressing phylogenetic questions and has greatly enhanced our understanding of the evolution in plants [50, 51]. The relationships between different *P. palustre* have not yet been reported thus it is essential to explore *P. palustre* diversity and evolution. In this study, combined with the cytotype, CP and SNP of the nuclear genome (Fig. 6), we determined that Guangdong occurred in all three ploidies accessions, which revealed that frequent polyploidization events occurred in *P. palustre*. Therefore, these different ploidy accessions from Guangdong are ideal materials for further exploration of polyploidization evolution. Thus, the observed phylogenetic inconsistencies and mixed ploidy levels may provide new clues about the origin of different *P. palustre* and the formation of polyploids from diploids. However, in this study, haplotype networks could not be conducted as the limited numbers of *P. palustre* accession, while it will be interesting for investigating the genealogical relationships among haplotypes in the future.

## Conclusions

We conducted FISH to identify *P. palustre* ploidy using 35 S and 5 S rDNA probes, and determined that 30 *P. palustre* accessions included three distinct cytotypes: diploid (2*n* = 2*x* = 30), triploid (2*n* = 3*x* = 45), and tetraploid (2*n* = 4*x* = 60). Illumina sequencing and CP genome assembly indicated that different *P. palustre* accessions have a conserved single circular molecule with a length of 152,534 − 152,788 bp, comprising a LSC and SSC separated by two IRs. Phylogenetic trees, based on CP and nuclear genomes, showed that the 30 *P. palustre* accessions clustered together, thereby corresponding to the collection site. Moreover, the Fujian region had the most diploid accessions, indicating that *P. palustre* may spread from Fujian to other regions. Overall, these findings help facilitate a deeper understanding of the karyotype and evolution of this species and will contribute to the genome sequencing of *P. palustre* in future studies.

## Supplementary Information

Supplementary Material 1.

## Data Availability

The sequences of resequencing of thirty *P. palustre* been deposited in the NCBI database, and the accession number is PRJNA1242048. The chloroplast genome of *P. palustre* used for analysis available from the NCBI, and their accession numbers are uploaded as PV476670-PV476683, respectively.

## Abbreviations

FISH: Fluorescence *in situ* hybridization
rDNA: Ribosomal DNA
SSC: Small single copy
LSC: Large single copy
IR: Inverted repeat
TIM3+F+R4: Transitional substitution model with 3 substitution types, empirical base frequencies, and freeRate heterogeneity
PCR: Polymerase chain reaction
CP: Chloroplast
SNP: Single nucleotide polymorphism
PCA: Principal component analysis
