# Supplementary material for: Cytotype classification and genetic diversity of Platostoma palustre revealed by rDNA localization and chloroplast genome
Source: BMC Genomics. 2025 Oct 21;26:937. doi: 10.1186/s12864-025-12118-3 (PMC12538835; doi:10.1186/s12864-025-12118-3)
Supplement: Supplementary file 1 — Supplementary Material 1. [file 12864_2025_12118_MOESM1_ESM.docx]

**Table S1.** Chromosomes statistics and localization of rDNA in *P. palustre*.

| **Clones** | **Chromosomes** | **No. and location of 5S rDNA** | **No. and location of 35S rDNA** | **Clones** | **Chromosomes** | **No. and location of 5S rDNA** | **No. and location of 35S rDNA** |
| --- | --- | --- | --- | --- | --- | --- | --- |
| JXJA | 45 | 3, pericentromere | 3, terminal | GDMZ | 45 | 3, pericentromere | 3, terminal |
| FJSSM5 | 30 | 2, pericentromere | 2, terminal | GXPN4 | 60 | 4, pericentromere | 4, terminal |
| FJC | 30 | 2, pericentromere | 2, terminal | GDZC1 | 45 | 3, pericentromere | 3, terminal |
| JXJA2 | 45 | 3, pericentromere | 3, terminal | GXLS | 45 | 3, pericentromere | 3, terminal |
| FJLY | 30 | 2, pericentromere | 2, terminal | GDC2 | 30 | 2, pericentromere | 2, terminal |
| FJXB | 30 | 2, pericentromere | 2, terminal | GDMM7 | 60 | 4, pericentromere | 4, terminal |
| GXJX | 60 | 4, pericentromere | 4, terminal | GDMM4 | 60 | 4, pericentromere | 4, terminal |
| GXWZ | 45 | 3, pericentromere | 3, terminal | FJXB4 | 45 | 3, pericentromere | 3, terminal |
| AHBM | 45 | 3, pericentromere | 3, terminal | FJSS3 | 30 | 2, pericentromere | 2, terminal |
| GXPBJP | 60 | 4, pericentromere | 4, terminal | GDMM6 | 60 | 4, pericentromere | 4, terminal |
| GXPN1 | 60 | 4, pericentromere | 4, terminal | GDMM5 | 60 | 4, pericentromere | 4, terminal |
| YNC | 45 | 3, pericentromere | 3, terminal | FJWP | 45 | 3, pericentromere | 3, terminal |
| GXPN2 | 60 | 4, pericentromere | 4, terminal | GDMM3 | 45 | 3, pericentromere | 3, terminal |
| GDC | 45 | 3, pericentromere | 3, terminal | GDMM2 | 60 | 4, pericentromere | 4, terminal |
| TWC | 30 | 2, pericentromere | 2, terminal | GDMM1 | 45 | 3, pericentromere | 3, terminal |


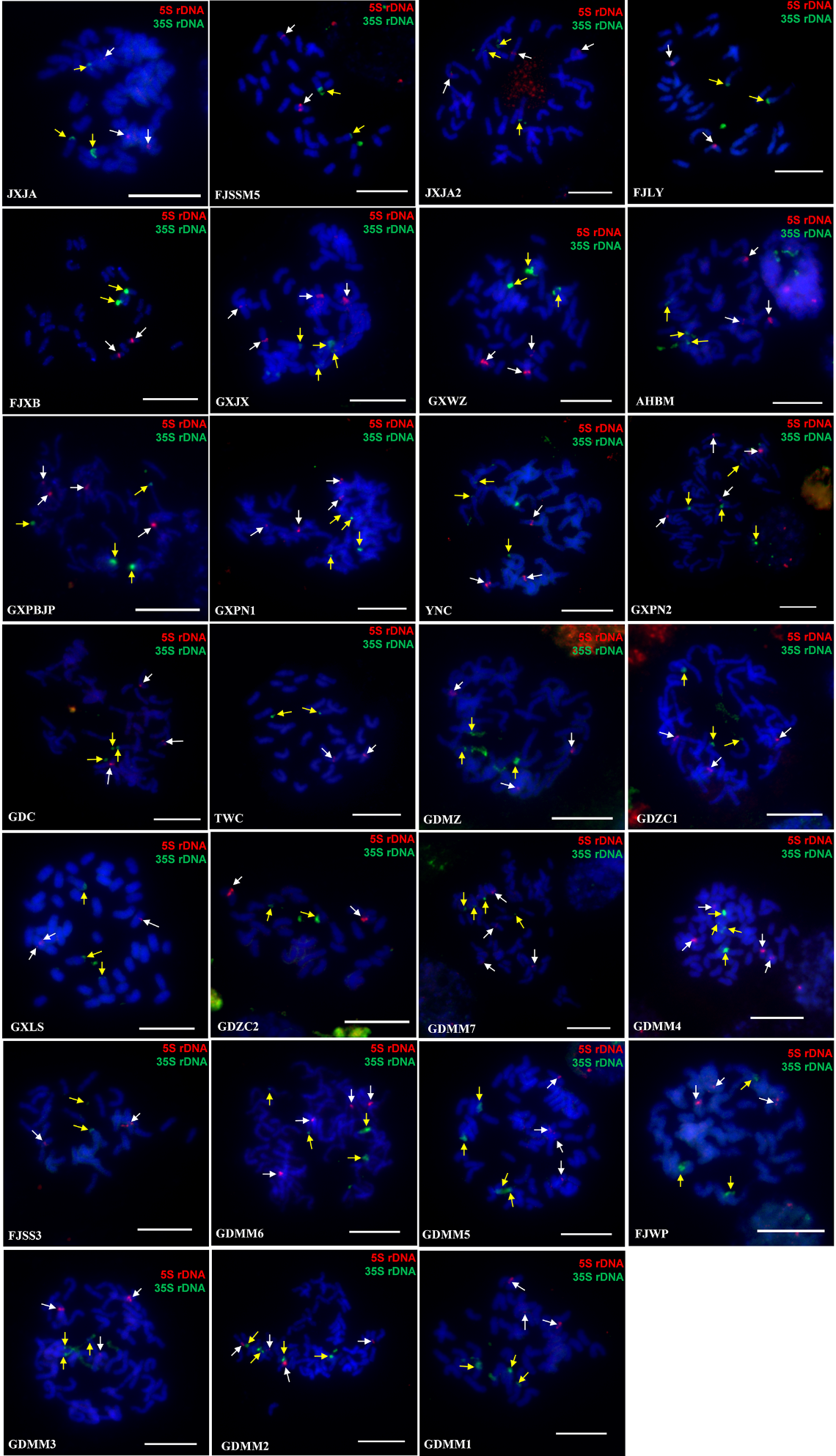


**Figure S1.** Cytogenetic analysis of 5S and 35S rDNAs on mitotic metaphase chromosomes of *P. palustre*. The 5S rDNA signals are green, and indicated by white arrows. 35S rDNA signals are red, and indicated by yellow arrows. Scale bars = 10 μm.

**Table S2.** Sequencing data statistics.

| **Clones** | **Raw reads** | **Raw bases** | **Clean reads** | **Clean bases** | **Q20** | **Q30** | **CG content** | **Reads passed filters** | **Average depth** |
| --- | --- | --- | --- | --- | --- | --- | --- | --- | --- |
| JXJA | 130,090,108 | 19,513,516,200 | 129,958,018 | 19,190,668,334 | 98.61% | 94.75% | 40.65% | 129,958,018 | 3.474 |
| FJSSM5 | 119,752,832 | 17,962,924,800 | 119,658,722 | 17,688,709,853 | 98.57% | 94.64% | 40.41% | 119,658,722 | 3.549 |
| FJC | 104,990,542 | 15,748,581,300 | 104,929,818 | 15,568,171,809 | 98.46% | 94.33% | 40.34% | 104,929,818 | 3.113 |
| JXJA2 | 121,977,486 | 18,296,622,900 | 121,844,998 | 18,097,461,748 | 98.28% | 93.55% | 43.08% | 121,844,998 | 3.17 |
| FJLY | 111,497,152 | 16,724,572,800 | 111,436,122 | 16,566,167,800 | 98.34% | 93.80% | 41.45% | 111,436,122 | 3.149 |
| FJXB | 137,670,608 | 20,650,591,200 | 137,577,882 | 20,333,342,958 | 98.47% | 94.42% | 40.74% | 137,577,882 | 3.642 |
| GXJX | 129,677,106 | 19,451,565,900 | 129,592,038 | 19,225,597,761 | 98.48% | 94.38% | 40.27% | 129,592,038 | 3.591 |
| GXWZ | 135,487,612 | 20,323,141,800 | 135,394,664 | 20,041,483,076 | 98.41% | 94.04% | 40.37% | 135,394,664 | 3.667 |
| AHBM | 92,534,902 | 13,880,235,300 | 92,418,312 | 13,692,560,991 | 98.29% | 93.51% | 40.45% | 92,418,312 | 2.713 |
| GXPBJP | 116,527,186 | 17,479,077,900 | 116,450,042 | 17,309,673,149 | 98.35% | 93.98% | 40.20% | 116,450,042 | 3.321 |
| GXPN1 | 133,081,148 | 19,962,172,200 | 132,960,910 | 19,638,388,154 | 98.44% | 94.21% | 40.23% | 132,960,910 | 3.432 |
| YNC | 117,881,374 | 17,682,206,100 | 117,796,324 | 17,509,031,159 | 98.38% | 93.95% | 40.34% | 117,796,324 | 3.439 |
| GXPN2 | 109,346,318 | 16,401,947,700 | 109,269,180 | 16,222,311,488 | 98.27% | 93.54% | 39.91% | 109,269,180 | 2.801 |
| GDC | 92,165,630 | 13,824,844,500 | 92,107,912 | 13,646,260,484 | 98.45% | 94.21% | 40.10% | 92,107,912 | 2.614 |
| TWC | 80,008,480 | 12,001,272,000 | 79,932,180 | 11,827,329,442 | 98.47% | 94.25% | 40.88% | 79,932,180 | 2.35 |
| GDMZ | 128,453,246 | 19,267,986,900 | 128,367,860 | 18,795,164,237 | 98.51% | 94.34% | 39.97% | 128,367,860 | 3.7 |
| GXPN4 | 127,217,556 | 19,082,633,400 | 127,129,930 | 18,645,475,367 | 98.42% | 94.06% | 40.44% | 127,129,930 | 3.413 |
| GDZC1 | 116,629,894 | 17,494,484,100 | 116,564,402 | 17,313,668,761 | 98.47% | 94.41% | 39.91% | 116,564,402 | 3.145 |
| GXLS | 128,907,596 | 19,336,139,400 | 128,816,850 | 19,065,260,542 | 98.46% | 94.34% | 40.12% | 128,816,850 | 3.934 |
| GDZC1 | 139,384,692 | 20,907,703,800 | 139,268,838 | 20,682,238,922 | 98.28% | 93.47% | 39.86% | 139,268,838 | 3.61 |
| GDMM7 | 127,344,712 | 19,101,706,800 | 127,256,968 | 18,880,896,121 | 98.32% | 93.68% | 40.28% | 127,256,968 | 3.446 |
| GDMM4 | 113,629,702 | 17,044,455,300 | 113,533,326 | 16,725,401,348 | 98.50% | 94.34% | 39.93% | 113,533,326 | 2.909 |
| FJXB4 | 97,658,360 | 14,648,754,000 | 97,598,950 | 14,494,451,247 | 98.34% | 93.79% | 39.99% | 97,598,950 | 3.279 |
| FJSS3 | 115,036,396 | 17,255,459,400 | 114,924,204 | 17,010,539,045 | 98.32% | 93.66% | 40.07% | 114,924,204 | 3.222 |
| GDMM6 | 108,733,758 | 16,310,063,700 | 108,632,974 | 15,945,892,225 | 98.24% | 93.34% | 40.10% | 108,632,974 | 2.966 |
| GDMM5 | 115,365,066 | 17,304,759,900 | 115,292,300 | 17,141,916,237 | 98.47% | 94.34% | 39.75% | 115,292,300 | 3.452 |
| FJWP | 119,594,480 | 17,939,172,000 | 119,508,126 | 17,743,232,282 | 98.59% | 94.83% | 39.84% | 119,508,126 | 3.661 |
| GDMM3 | 89,674,290 | 13,451,143,500 | 89,624,788 | 13,314,533,958 | 98.58% | 94.77% | 40.10% | 89,624,788 | 2.545 |
| GDMM2 | 105,610,156 | 15,841,523,400 | 105,540,386 | 15,668,930,335 | 98.54% | 94.60% | 39.87% | 105,540,386 | 2.783 |
| GDMM1 | 109,104,606 | 16,365,690,900 | 109,020,194 | 16,133,814,941 | 98.56% | 94.67% | 39.55% | 109,020,194 | 3.173 |

**Table S3.** The length of assembly CP genome in *P. palustre*.

| **Clones** | **Group** | **The length of cp (bp)** | **The length of IR (bp)** | **The length of LSC (bp)** | **The length of SSC (bp)** |
| --- | --- | --- | --- | --- | --- |
| FJC, FJXB, TWC | I | 152,534 | 51,336 | 83,450 | 17,748 |
| GDMM7, GDMM2 | II | 152,555 | 51,340 | 83,490 | 17,725 |
| GXPN4, GDMM5, GXPN2 | III | 152,635 | 51,370 | 83,514 | 17,751 |
| GXPBJP | IV | 152,681 | 51,334 | 83,571 | 17,776 |
| AHBM, GXLS, FJWP, GDZC2 | V | 152,547 | 51,340 | 83,482 | 17,725 |
| GDZC1, GDMZ, GXWZ, GDC | VI | 152,556 | 51,340 | 83,491 | 17,725 |
| GXJX, YNC, GXPN1, GDMM3 | VII | 152,635 | 51,370 | 83,514 | 17,751 |
| JXJA2 | VIII | 152,644 | 51,370 | 83,522 | 17,752 |
| SSM5, FJLY, SS3 | VIIII | 152,535 | 51,336 | 83,451 | 17,748 |
| JXJA | X | 152,643 | 51,370 | 83,522 | 17,751 |
| GDMM1 | XI | 152,788 | 51,774 | 83,301 | 17,743 |
| GDMM6 | XII | 152,786 | 51,774 | 83,300 | 17,742 |
| FJXB4 | XIII | 152,534 | 51,336 | 83,450 | 17,748 |
| GXPN4, GDMM5, GXPN2 | XIIII | 152,559 | 51,340 | 83,494 | 17,725 |

**Table S4.** Gene composition in *P. palustre* CP genome.

| **Category of genes** | **Group of genes** | **Name of genes** |
| --- | --- | --- |
| Genes for photosynthesis | Subunits of ATP synthase | atpA, atpB, atpE, atpF, atpH, atpI |
|  | Subunits of photosystem II | psbA, psbB, psbC, psbD, psbE, psbF, psbI, psbJ, psbK, psbL, psbM, psbN, psbT, psbZ, ycf3 |
|  | Subunits of NADH-dehydrogenase | ndhA, ndhB, ndhB, ndhC, ndhD, ndhE, ndhF, ndhG, ndhH, ndhI, ndhJ, ndhK |
|  | Subunits of cytochrome b/f complex | petA, petB, petD, petG, petL, petN |
|  | Subunits of photosystem I | psaA, psaB, psaC, psaI, psaJ |
|  | Subunit of rubisco | rbcL |
| Self replication | Large subunit of ribosome | rpl14, rpl16, rpl2, rpl2, rpl20, rpl22, rpl23, rpl23, rpl32, rpl33, rpl36 |
|  | DNA dependent RNA polymerase | rpoA, rpoB, rpoC1, rpoC2 |
|  | Small subunit of ribosome | rps11, rps12, rps12, rps14, rps15, rps16, rps18, rps19, rps2, rps3, rps4, rps7, rps7, rps8 |
| Other genes | Subunit of Acetyl-CoA-carboxylase | accD |
|  | c-type cytochrom synthesis gene | ccsA |
|  | Envelop membrane protein | cemA |
|  | Protease | clpP |
|  | Maturase | matK |
| Unkown | Conserved open reading frames | ycf1, ycf1, ycf2, ycf2, ycf4 |


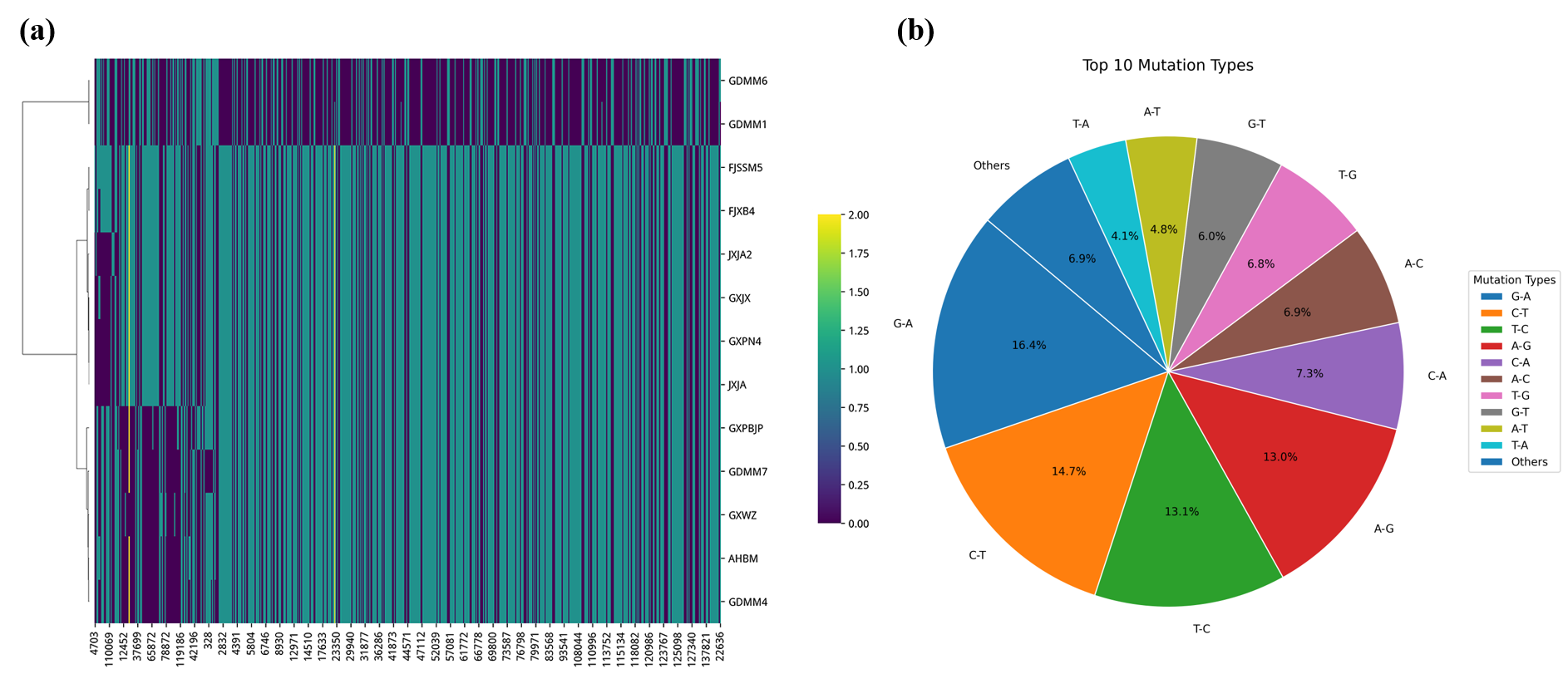


**Figure S2.** The SNP variation of the chloroplast genome of *P. palustre*. **(a)** SNP density distribution on the chloroplast genome with FJC as a reference. **(b)** Types of base mutations in *P. palustre*.
